# Publisher Correction: Re-analysis of public genetic data reveals a rare X-chromosomal variant associated with type 2 diabetes

**DOI:** 10.1038/s41467-018-04170-3

**Published:** 2018-05-30

**Authors:** Sílvia Bonàs-Guarch, Marta Guindo-Martínez, Irene Miguel-Escalada, Niels Grarup, David Sebastian, Elias Rodriguez-Fos, Friman Sánchez, Mercè Planas-Fèlix, Paula Cortes-Sánchez, Santi González, Pascal Timshel, Tune H. Pers, Claire C. Morgan, Ignasi Moran, Goutham Atla, Juan R. González, Montserrat Puiggros, Jonathan Martí, Ehm A. Andersson, Carlos Díaz, Rosa M. Badia, Miriam Udler, Aaron Leong, Varindepal Kaur, Jason Flannick, Torben Jørgensen, Allan Linneberg, Marit E. Jørgensen, Daniel R. Witte, Cramer Christensen, Ivan Brandslund, Emil V. Appel, Robert A. Scott, Jian’an Luan, Claudia Langenberg, Nicholas J. Wareham, Oluf Pedersen, Antonio Zorzano, Jose C Florez, Torben Hansen, Jorge Ferrer, Josep Maria Mercader, David Torrents

**Affiliations:** 1 Barcelona Supercomputing Center (BSC), Joint BSC-CRG-IRB Research Program in Computational Biology, 08034 Barcelona, Spain; 2 Genomic Programming of Beta-cells Laboratory, Institut d’Investigacions August Pi i Sunyer (IDIBAPS), 08036 Barcelona, Spain; 3 Instituto de Salud Carlos III, Centro de Investigación Biomédica en Red de Diabetes y Enfermedades Metabólicas Asociadas (CIBERDEM), 28029 Madrid, Spain; 4 Section of Epigenomics and Disease, Department of Medicine, Imperial College London, London, W12 0NN UK; 5 The Novo Nordisk Foundation Center for Basic Metabolic Research, Section for Metabolic Genetics, Faculty of Health and Medical Sciences, University of Copenhagen, 2100 Copenhagen, Denmark; 6 Institute for Research in Biomedicine (IRB Barcelona), The Barcelona Institute of Science and Technology, Baldiri Reixac 10-12, 08028 Barcelona, Spain; 7 Departament de Bioquímica i Biomedicina Molecular, Facultat de Biologia, Universitat de Barcelona, 08028 Barcelona, Spain; 8 Computer Sciences Department, Barcelona Supercomputing Center (BSC-CNS), 08034 Barcelona, Spain; 9 Department of Epidemiology Research, Statens Serum Institut, 2300 Copenhagen, Denmark; 10 Division of Endocrinology and Center for Basic and Translational Obesity Research, Boston Children’s Hospital, Boston, MA 02116 USA; 11 Medical and Population Genetics Program, Broad Institute of MIT and Harvard, Cambridge, MA 02142 USA; 12 ISGlobal, Centre for Research in Environmental Epidemiology (CREAL), 08003 Barcelona, Spain; 13 CIBER Epidemiología y Salud Pública (CIBERESP), 28029 Madrid, Spain; 14 Universitat Pompeu Fabra (UPF), 08003 Barcelona, Spain; 15 Artificial Intelligence Research Institute (IIIA), Spanish Council for Scientific Research (CSIC), 28006 Madrid, Spain; 16 Programs in Metabolism and Medical and Population Genetics, Broad Institute of Harvard and MIT, Cambridge, MA 02142 USA; 17 Diabetes Unit and Center for Genomic Medicine, Massachusetts General Hospital, Boston, MA 02114 USA; 18 Division of General Internal Medicine, Massachusetts General Hospital, Boston, MA 02114 USA; 19 Department of Molecular Biology, Harvard Medical School, Boston, MA 02114 USA; 20 Research Centre for Prevention and Health, Capital Region of Denmark, DK-2600 Glostrup, Denmark; 21 Faculty of Health and Medical Sciences, University of Copenhagen, DK-2200 Copenhagen, Denmark; 22 Faculty of Medicine, University of Aalborg, DK-9220 Aalborg East, Denmark; 23 Department of Clinical Experimental Research, Rigshospitalet, Glostrup, 2100 Copenhagen, Denmark; 24 Department of Clinical Medicine, Faculty of Health and Medical Sciences, University of Copenhagen, DK-2200 Copenhagen, Denmark; 25 Steno Diabetes Center, 2820 Gentofte, Denmark; 26 National Institute of Public Health, Southern Denmark University, DK-5230 Odense M, Denmark; 27 Department of Public Health, Aarhus University, DK-8000 Aarhus C, Denmark; 28 Danish Diabetes Academy, DK-5000 Odense C, Denmark; 29 Medical department, Lillebaelt Hospital, 7100 Vejle, Denmark; 30 Department of Clinical Biochemistry, Lillebaelt Hospital, 7100 Vejle, Denmark; 31 Institute of Regional Health Research, University of Southern Denmark, DK-5230 Odense, Denmark; 32 MRC Epidemiology Unit, University of Cambridge School of Clinical Medicine, Cambridge Biomedical Campus, Cambridge, CB2 0QQ UK; 33 Department of Medicine, Harvard Medical School, Boston, MA 02115 USA; 34 Faculty of Health Sciences, University of Southern Denmark, DK-5230 Odense M, Denmark; 35 Institució Catalana de Recerca i Estudis Avançats (ICREA), 08010 Barcelona, Spain

Correction to: *Nature Communications* 10.1038/s41467-017-02380-9, published online 22 January 2018

In the originally published version of this Article, the affiliation details for Santi González, Jian’an Luan and Claudia Langenberg were inadvertently omitted. Santi González should have been affiliated with 'Barcelona Supercomputing Center (BSC), Joint BSC-CRG-IRB Research Program in Computational Biology, 08034 Barcelona, Spain’, and Jian’an Luan and Claudia Langenberg should have been affiliated with ‘MRC Epidemiology Unit, University of Cambridge School of Clinical Medicine, Cambridge Biomedical Campus, Cambridge CB2 0QQ, UK’. Furthermore, the abstract contained an error in the SNP ID for the rare variant in chromosome Xq23, which was incorrectly given as rs146662057 and should have been rs146662075. These errors have now been corrected in both the PDF and HTML versions of the Article.

