# Supplementary material for: Re-analysis of public genetic data reveals a rare X-chromosomal variant associated with type 2 diabetes
Source: Nat Commun. 2018 Jan 22;9:321. doi: 10.1038/s41467-017-02380-9 (PMC5778074; doi:10.1038/s41467-017-02380-9)
Supplement: Supplementary file 1 — Supplementary Information [file 41467_2017_2380_MOESM1_ESM.pdf]

Supplementary Information

Supplementary Figures

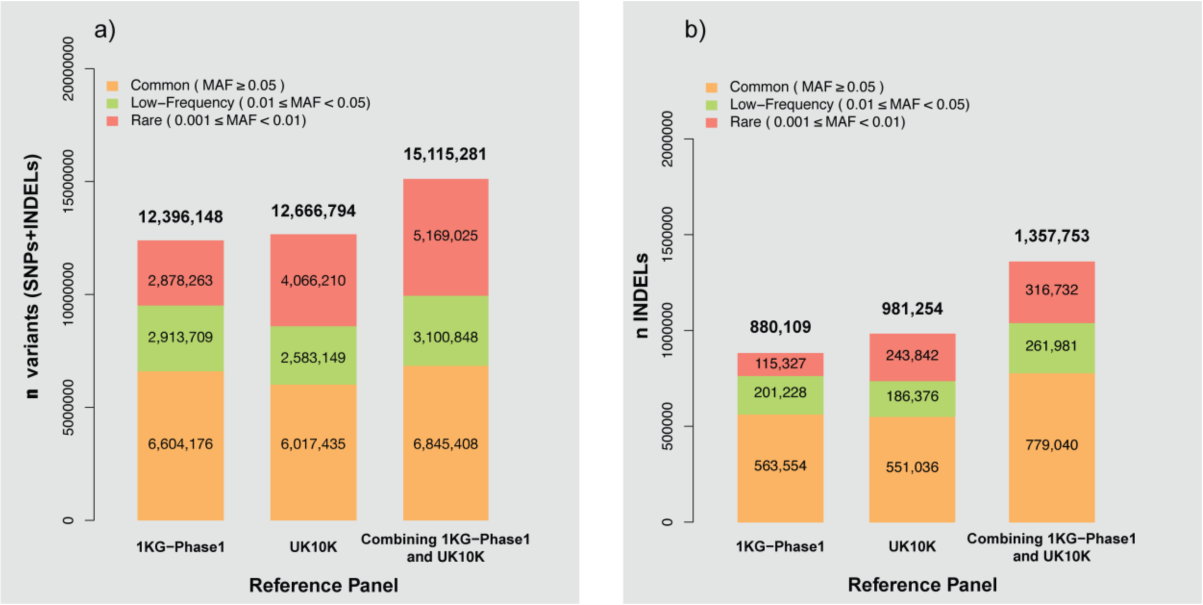

Supplementary Fig. 1. Improvement of genomic resolution from genotype imputation based on multiple reference panels compared to only using a single reference panel. a) All variants. b) Insertion and deletions. Each bar represents the genomic coverage from the final meta-analysis for the 70KforT2D cohort according to the reference panel used: from left to right, (1) 1000G-Phase1 release, (2) UK10K and (3) when combining the best-imputed results from 1000G-Phase1 and UK10K reference panels. Each bar was stratified according to the range of allele frequency: rare variants (0.001 ≤ MAF < 0.01), low frequency variants (0.01 ≤ MAF < 0.05) and common variants (MAF ≥ 0.05). Y-axis represents the absolute number of variants that passed all post-imputation quality filters, including IMPUTE2 info score ≥ 0.7.

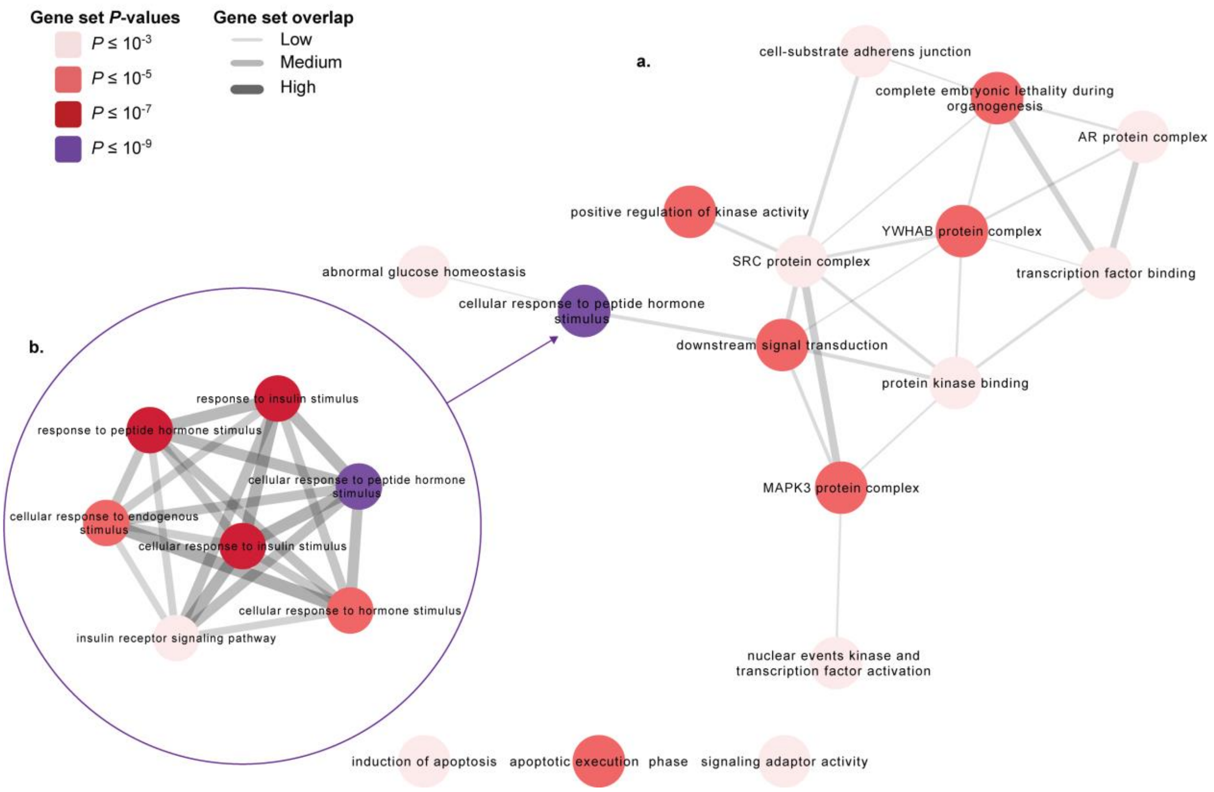

Supplementary Fig. 2. Network plot representing all significantly enriched cluster pathways (FDR<5%) using DEPICT. As input, we used all summary statistics with $p \leq 1 \times 10^{-5}$ in the 70KforT2D meta-analysis. Significantly enriched pathways were clustered by merging all pathways showing correlation higher than 0.3 into a single cluster using the affinity Propagation tool. The dependency between clusters is represented by the width of the edges. An expanded version of the "cellular response to peptide hormone stimulus" cluster is represented, showing all the pathways that showed enrichment within this cluster and their dependencies.

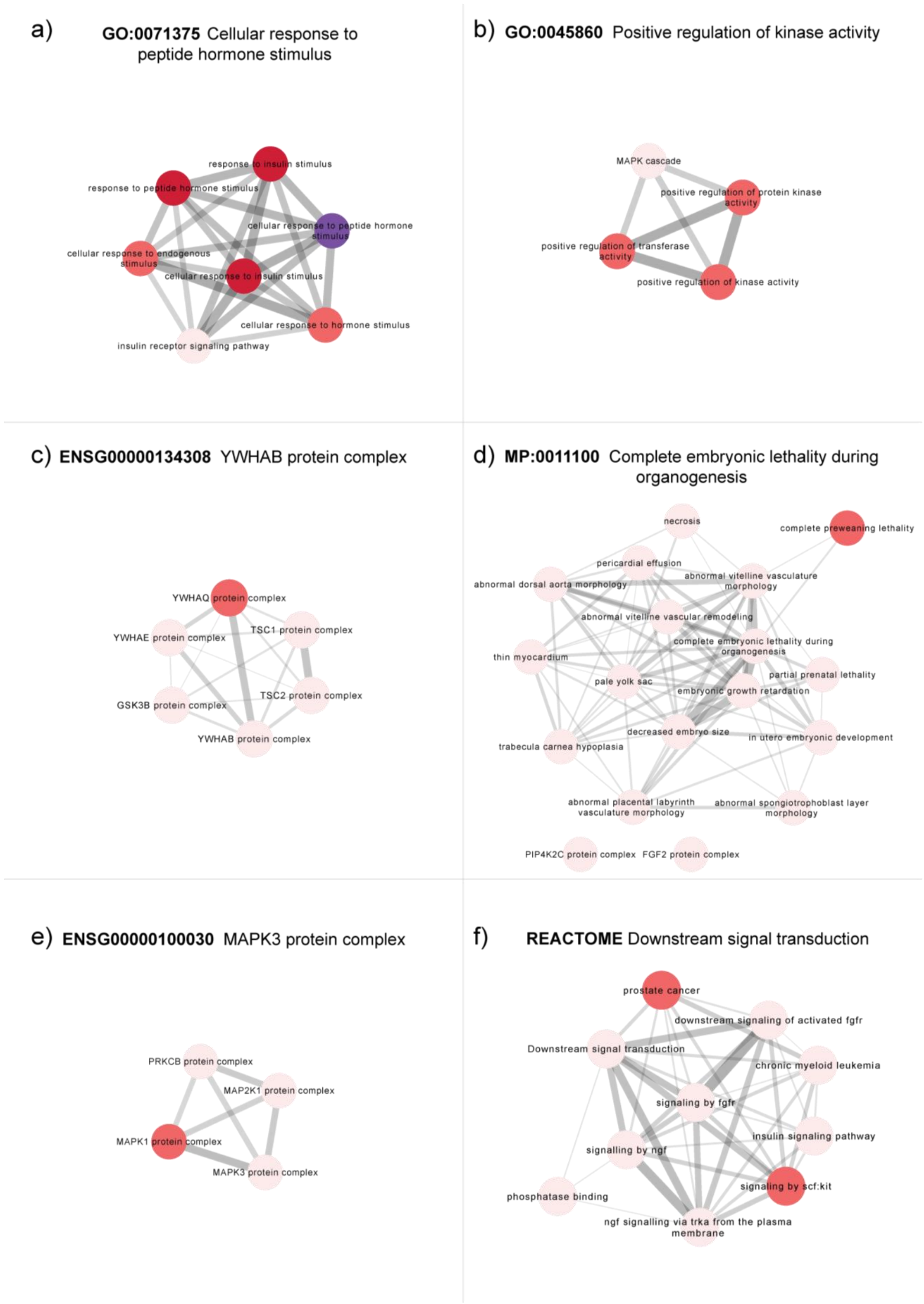
a) GO:0071375 Cellular response to peptide hormone stimulus
response to insulin stimulus
response to peptide hormone stimulus
cellular response to peptide hormone stimulus
cellular response to endogenous stimulus
cellular response to insulin stimulus
cellular response to hormone stimulus
insulin receptor signaling pathway
b) GO:0045860 Positive regulation of kinase activity
MAPK cascade
positive regulation of protein kinase activity
positive regulation of transferase activity
positive regulation of kinase activity
c) ENSG00000134308 YWHAB protein complex
YWHAQ protein complex
YWHAE protein complex
TSC1 protein complex
GSK3B protein complex
TSC2 protein complex
YWHAB protein complex
d) MP:0011100 Complete embryonic lethality during organogenesis
necrosis
complete preweaning lethality
pericardial effusion
abnormal dorsal aorta morphology
abnormal vitelline vasculature morphology
abnormal vitelline vascular remodeling
complete embryonic lethality during organogenesis
thin myocardium
partial prenatal lethality
pale yolk sac
embryonic growth retardation
decreased embryo size
in utero embryonic development
trabecula carnea hypoplasia
abnormal placental labyrinth vasculature morphology
abnormal spongiotrophoblast layer morphology
PIP4K2C protein complex
FGF2 protein complex
e) ENSG00000100030 MAPK3 protein complex
PRKCB protein complex
MAP2K1 protein complex
MAPK1 protein complex
MAPK3 protein complex
f) REACTOME Downstream signal transduction
prostate cancer
downstream signaling of activated fgfr
Downstream signal transduction
chronic myeloid leukemia
signaling by fgfr
insulin signaling pathway
signalling by ngf
signaling by scf:kit
phosphatase binding
ngf signalling via trka from the plasma membrane

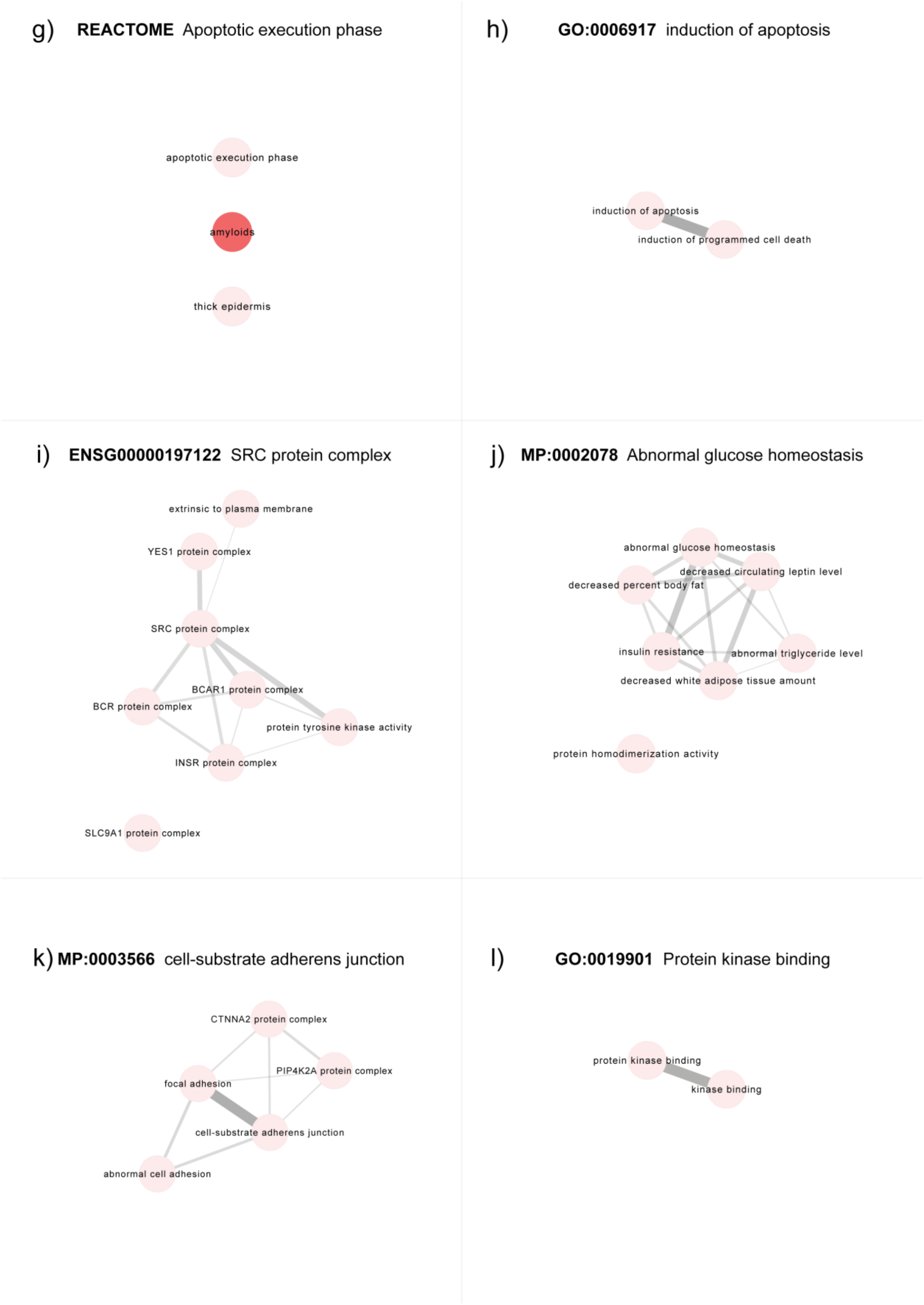
g) REACTOME Apoptotic execution phase
apoptotic execution phase
amyloids
thick epidermis
h) GO:0006917 induction of apoptosis
induction of apoptosis
induction of programmed cell death
i) ENSG00000197122 SRC protein complex
extrinsic to plasma membrane
YES1 protein complex
SRC protein complex
BCAR1 protein complex
BCR protein complex
protein tyrosine kinase activity
INSR protein complex
SLC9A1 protein complex
j) MP:0002078 Abnormal glucose homeostasis
abnormal glucose homeostasis
decreased circulating leptin level
decreased percent body fat
insulin resistance
abnormal triglyceride level
decreased white adipose tissue amount
protein homodimerization activity
k) MP:0003566 cell-substrate adherens junction
CTNNA2 protein complex
PIP4K2A protein complex
focal adhesion
cell-substrate adherens junction
abnormal cell adhesion
l) GO:0019901 Protein kinase binding
protein kinase binding
kinase binding

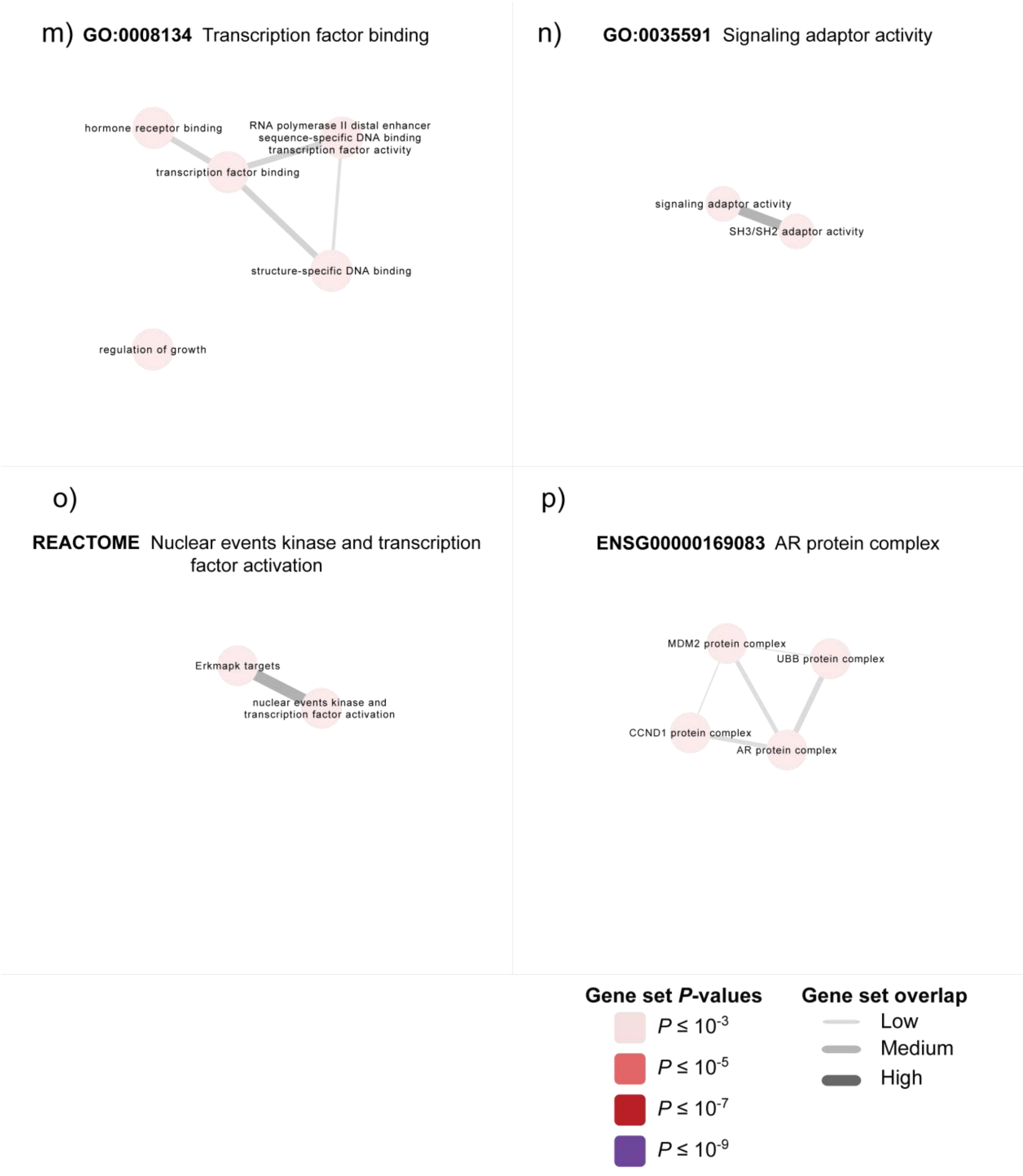

Supplementary Fig. 3. a)-p) Pathway analysis from GWAS results using DEPICT. Expanded representation of each of the network clusters that were significantly enriched (FDR<0.05). The correlation between each pathway is represented by the width of the edges.

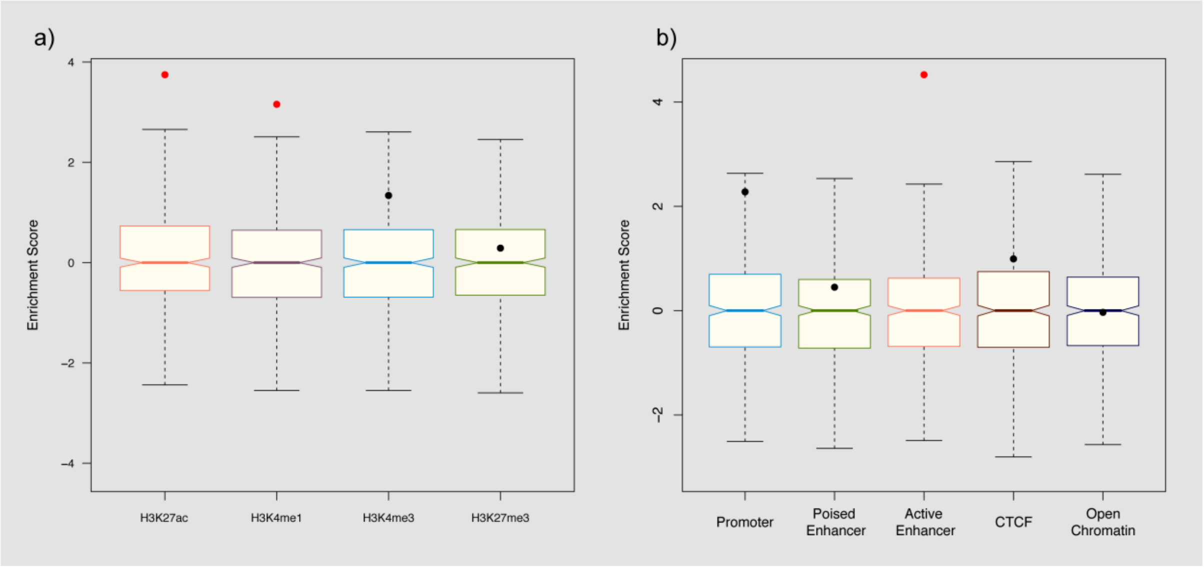

Supplementary Fig. 4. Variant Set Enrichment (VSE) analysis of 99% credible sets SNPs ($R^2 \geq 0.4$) of T2D associated regions. Enrichment for a) histone modifications and b) regulatory elements defined in isolated human pancreatic islets, as described by Pasquali, L. and colleagues[1]**.** Boxplots represent null distributions based on 500 match-random SNP sets and each dot shows the observed enrichment value relative to the null distribution. Dots highlighted in red achieved significance after applying Bonferroni correction (p<0.01).

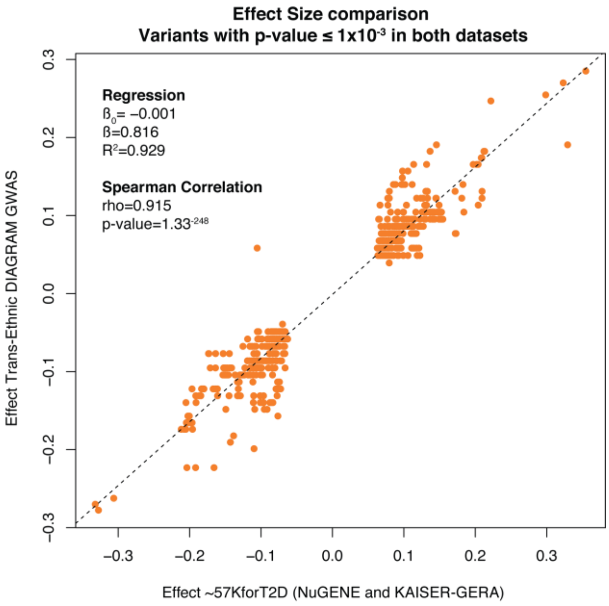

Supplementary Fig. 5. Comparison of log-odds ratios in the non-overlapping cohorts analysed and previously published results from DIAGRAM trans-ancestry meta-analysis. Each point represents the corresponding log-odds ratio. The analysis comprised all variants with $p<1\times10^{-3}$ in both datasets.

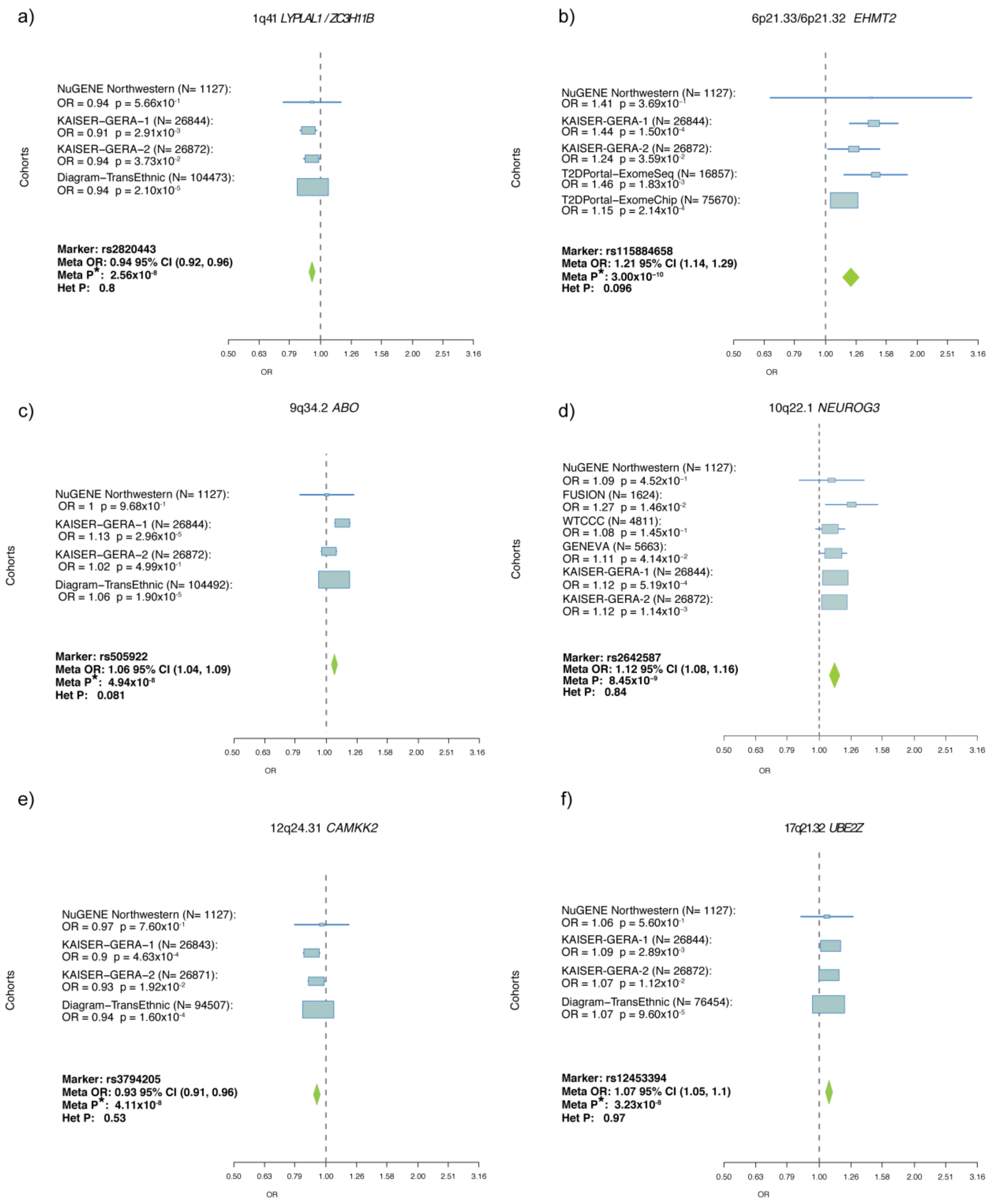

Supplementary Fig. 6. a)-f) Forest plots for the lead variants of each of the novel locus using data from the discovery and replication datasets, except the Xq23 chromosome locus. Cohort-specific odds ratios (95% CIs) are denoted by blue boxes (blue lines). The combined OR estimate for all the datasets is represented by a green diamond, where the diamond width corresponds to 95% CI bounds. The *p*-value for the meta-analysis (Meta P) and for the heterogeneity (Het P) of odds ratio is shown. The header of each forest plot represents the closest gene to the lead variant. **P*-values obtained with a sample size meta-analysis instead of a fixed effects inverse-variance meta-analysis.

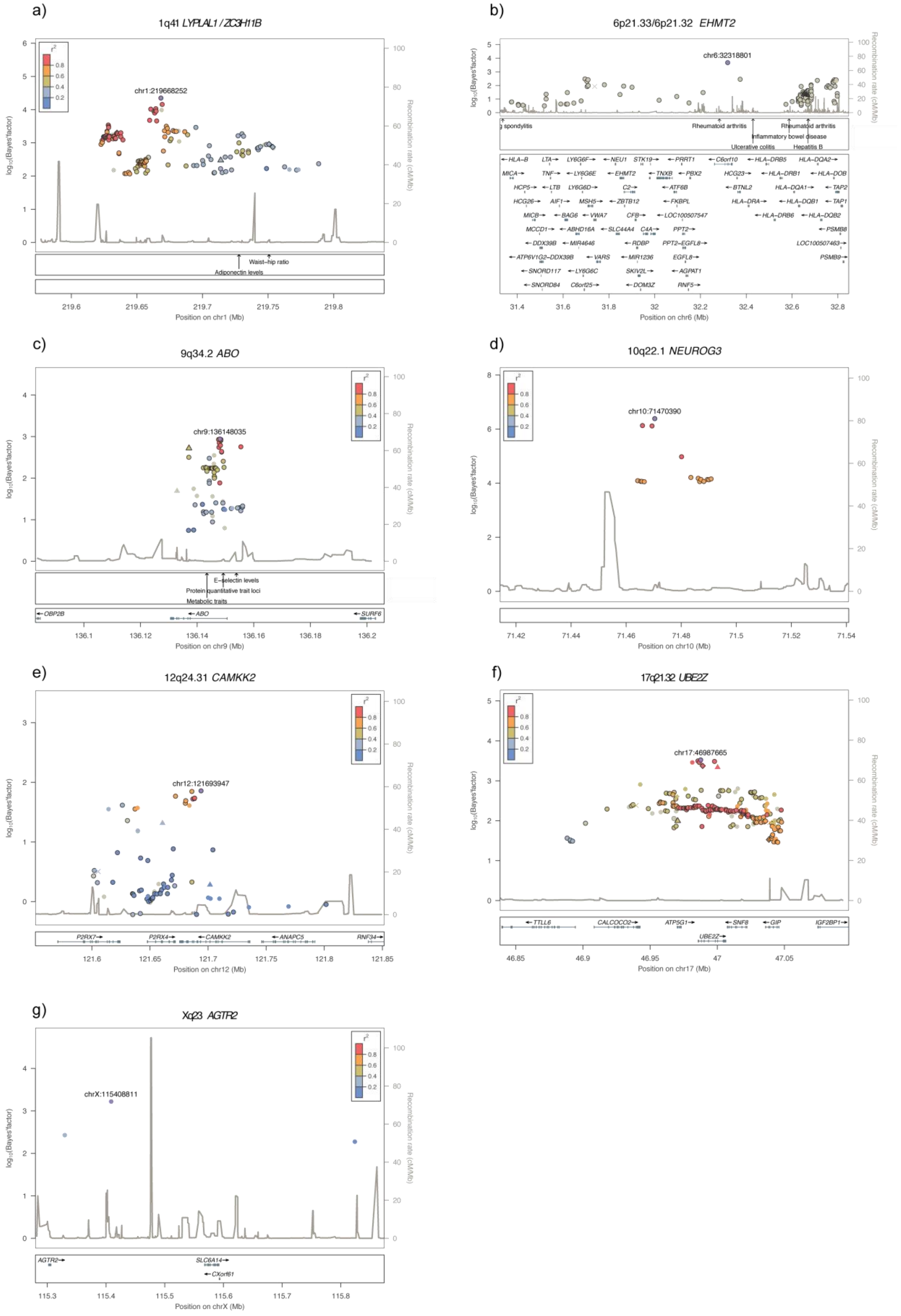

a)
1q41 LYPLAL1 / ZC3H11B
chr1:219668252
Waist−hip ratio
Adiponectin levels
Position on chr1 (Mb)
b)
6p21.33/6p21.32 EHMT2
chr6:32318801
Rheumatoid arthritis
Inflammatory bowel disease
Ulcerative colitis
Hepatitis B
Position on chr6 (Mb)
c)
9q34.2 ABO
chr9:136148035
E−selectin levels
Protein quantitative trait loci
Metabolic traits
OBP2B
ABO
SURF6
Position on chr9 (Mb)
d)
10q22.1 NEUROG3
chr10:71470390
Position on chr10 (Mb)
e)
12q24.31 CAMKK2
chr12:121693947
P2RX7
P2RX4
CAMKK2
ANAPC5
RNF34
Position on chr12 (Mb)
f)
17q21.32 UBE2Z
chr17:46987665
TTLL6
CALCOCO2
ATP5G1
SNF8
GIP
IGF2BP1
UBE2Z
Position on chr17 (Mb)
g)
Xq23 AGTR2
chrX:115408811
AGTR2
SLC6A14
CXorf61
Position on chrX (Mb)
log10(Bayes factor)
Recombination rate (cM/Mb)
r²

Supplementary Fig. 7. a)-g) Signal plots representing the 99% credible sets of SNPs at the 7 novel loci. In each plot, each point represents a variant within the 99% credible set with the Bayes' factor (y axis, on a $\log_{10}$ scale) as a function of genomic position (hg19). The lead SNP is represented by the purple symbol. The color-coding scheme indicates the R-squared with the lead SNP, estimated based on 1000G r^2 values from European population. Recombination rates were estimated from Phase II HapMap and gene annotations from the UCSC genome browser. Only the SNPs that fall within the 99% credible set are plotted.

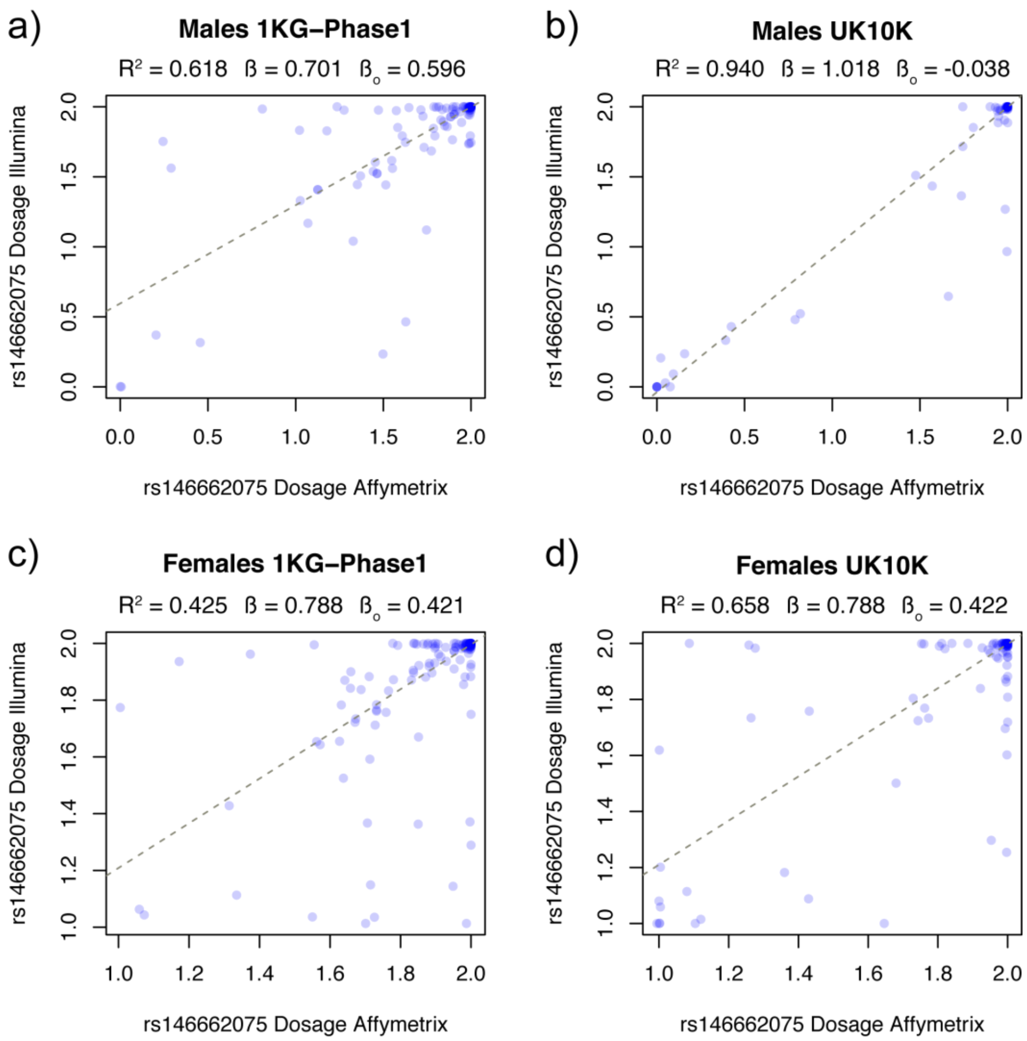

Supplementary Fig. 8. Comparison of imputation quality in males and females and UK10K and 1000 G phase1 reference panels. To evaluate genotype imputation quality, we imputed genotypes into the 58C cohort from the WTCCC, which consisted on a dataset of ~3,000 individuals that were genotyped by both Affymetrix v6.0 (Affy) and Illumina 1.2M (IL) platforms. We performed genotype imputation independently using either Affy or IL genotypes as the backbone. The quality of the imputed variants was evaluated using the allelic dosage R^2 coefficient (see Supplementary Methods) between the genotype dosages estimated when imputing using Affy or Illumina as backbone. We show the imputation results for a) males to 1000G and b) UK10K reference panels, and then, for c) females to 1000G and d) UK10K. Genotype imputation is of higher quality in males to UK10K.

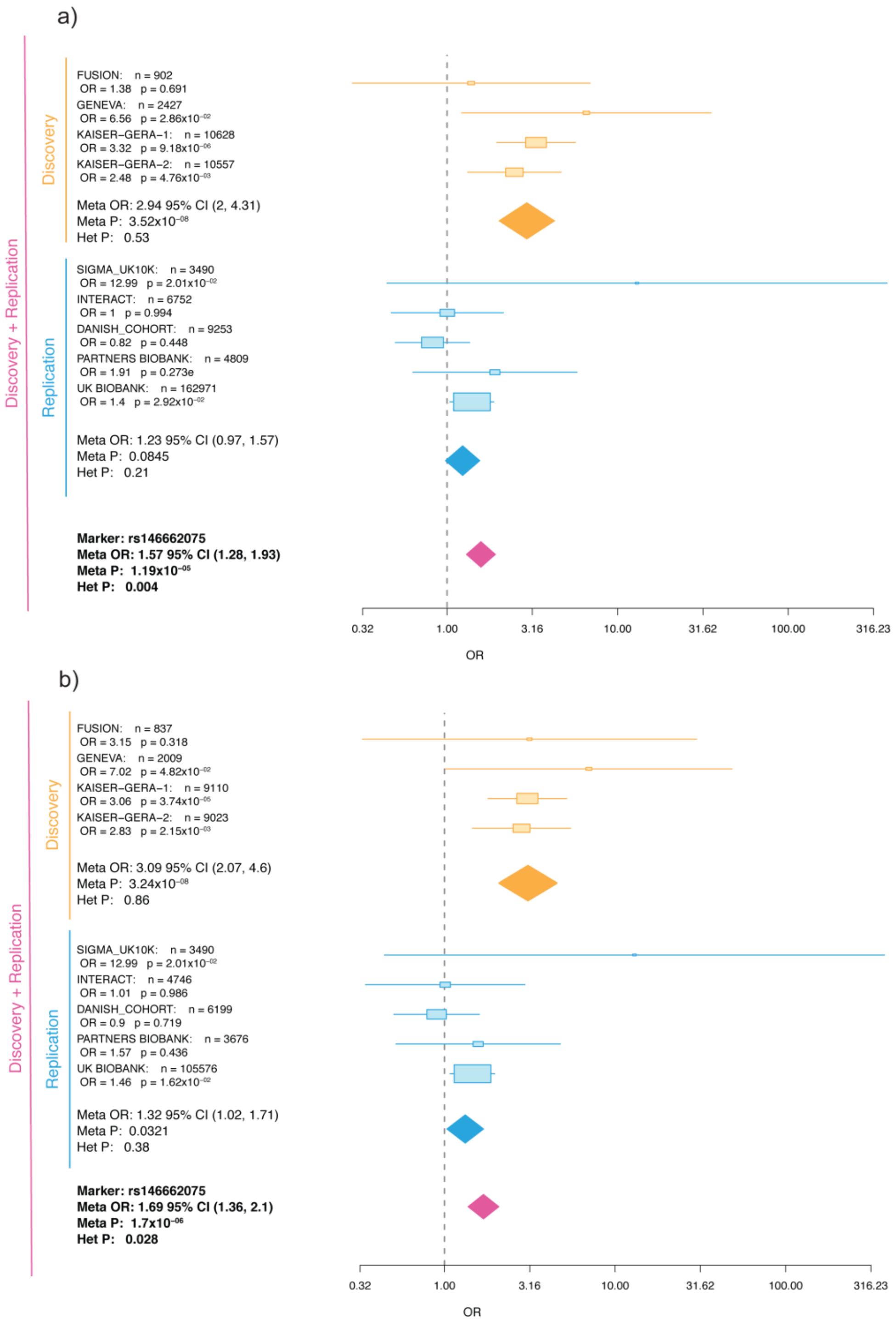

Supplementary Fig. 9. Discovery and replication of rs14666075 association signal. Forest plots for rs146662075 using data from the discovery and replication datasets when a) not applying any additional filter to the control samples, b) when only excluding controls younger than 55 years old. Cohort-specific odds ratios (95% CIs) are denoted by blue boxes (blue lines). The combined OR estimate for all the datasets is represented by a green diamond, where the diamond width corresponds to 95% CI bounds. The *p*-value for the meta-analysis (Meta P) and for the heterogeneity (Het P) of odds ratio is shown.

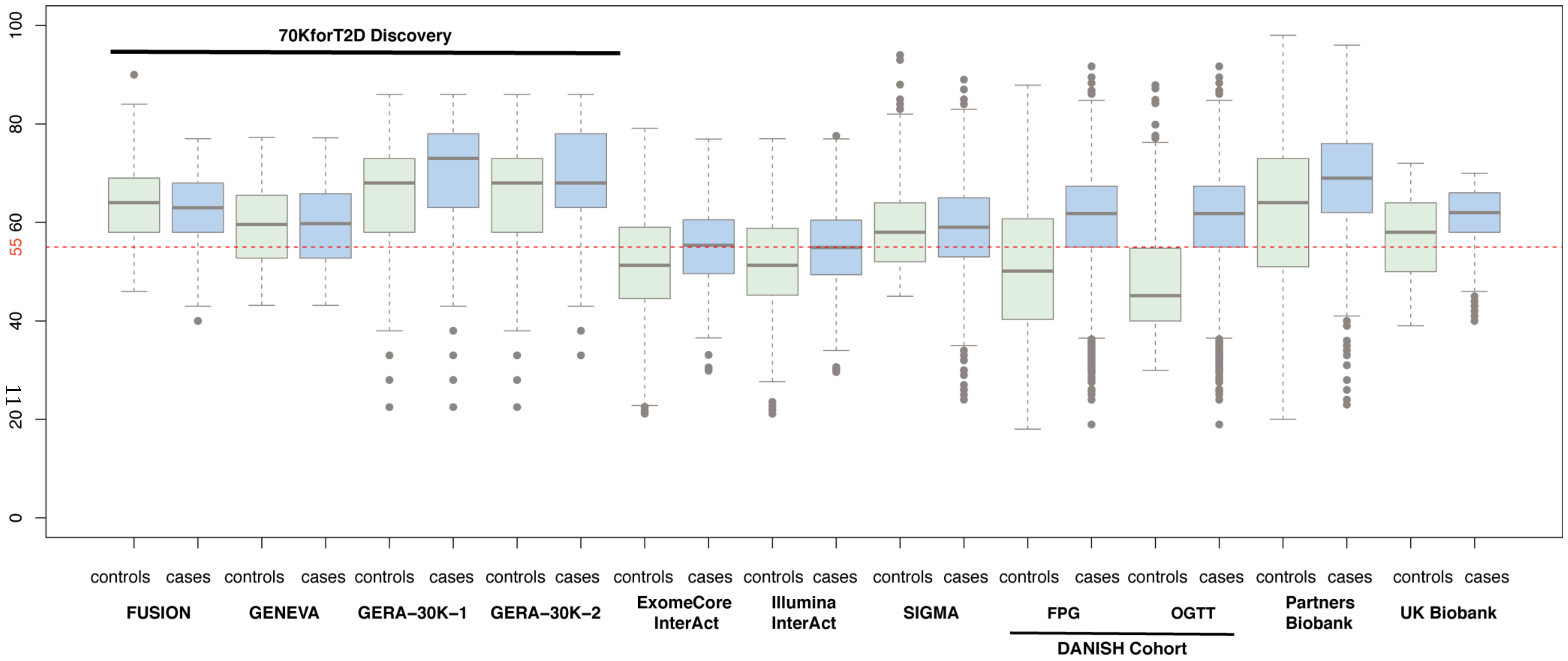
100
80
60
55
40
20
0
70KforT2D Discovery
controls
cases
FUSION
GENEVA
GERA–30K–1
GERA–30K–2
ExomeCore InterAct
Illumina InterAct
SIGMA
FPG
OGTT
DANISH Cohort
Partners Biobank
UK Biobank

Supplementary Fig. 10. Boxplot representing the distribution of ages in cases and controls across cohorts. The red line represents 55 years old, which is the average age at onset of T2D in European populations.

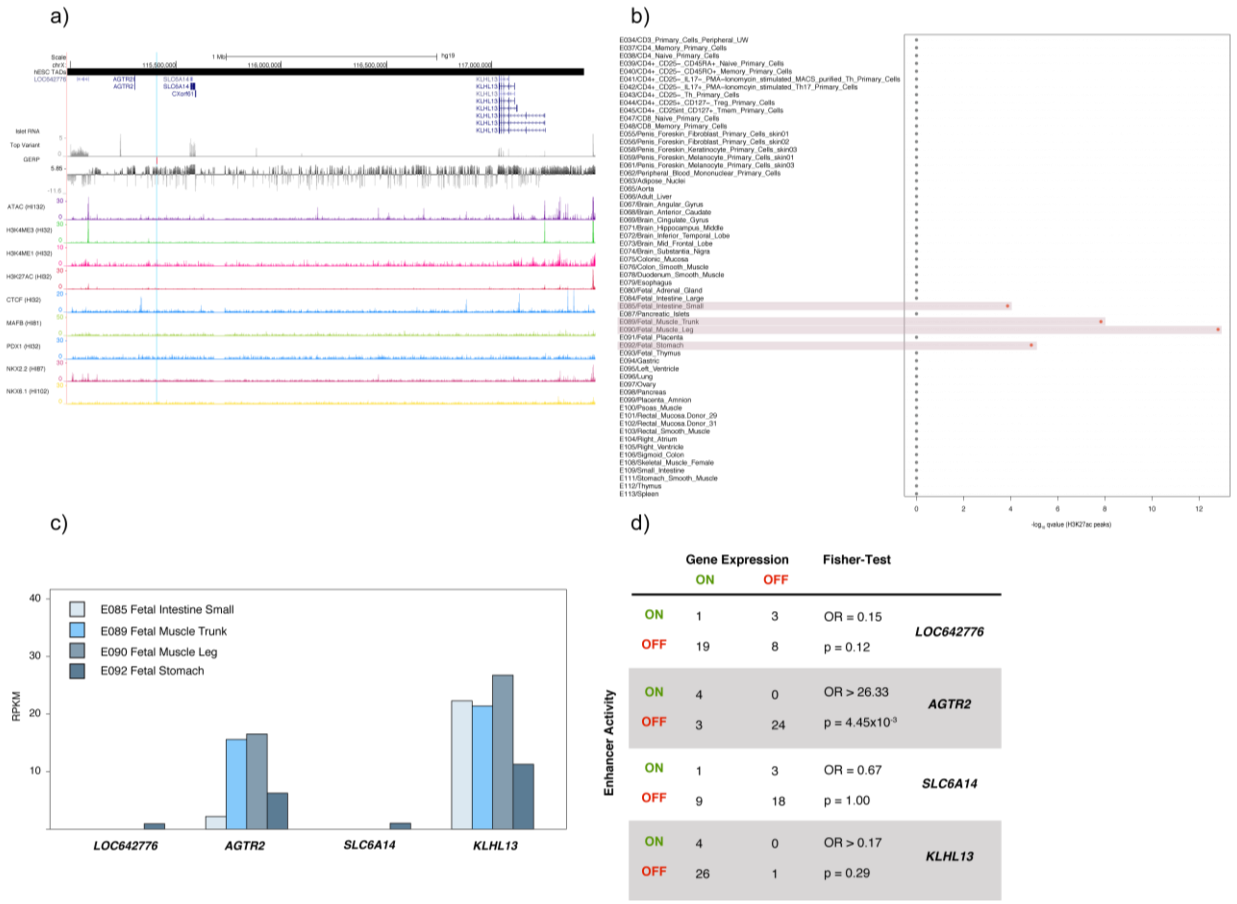

Supplementary Fig. 11. Characterizing the transcriptional regulatory activity of the rs146662075 enhancer element. a) UCSC screenshot showing representative ChIP-Seq datasets for transcription binding and chromatin marks associated to active enhancer elements in human islets within the TAD domain in which rs146662075 is located (highlighted in blue). b) Representation of the rs14666075 enhancer activity according to the $-\log_{10}$ MACS2 *q*-value from H3K27ac narrow peaks across multiple tissues from the NIH Roadmap Epigenomics Mapping Consortium consolidated epigenomes dataset. c) Gene expression levels for candidate target genes of the rs146662075 enhancer variant in those tissues in which significant enhancer activity was observed in b). d) Association between enhancer activity and gene expression for each of the candidate target genes. For each candidate target gene, a contingency table showing the tissue's counts is represented for each of the 4 scenarios and the estimate odds ratio (OR) and the *p*-value from the Fisher's Exact Test is also provided.

Supplementary Note 1

Details of independent discovery GWAS datasets

We collected all publicly genetic individual-level data for Type 2 diabetes (T2D) case/control studies from 5 independent datasets available in the dbGaP (http://www.ncbi.nlm.nih.gov/gap) and EGA (https://www.ebi.ac.uk/ega/home) public repositories, comprising a total of 13,201 cases and 59,656 controls. Each dataset was independently harmonized and quality controlled before performing genotype imputation and association testing.

NuGENE NORTHWESTERN

dbGaP Study Accession: phs000237.v1.p1
Ethnicity: European (USA)
T2D cases after QC: 527
Controls after QC: 601

Type 2 diabetes case selection criteria:

Neither group should have T1D diagnosis codes (ICD-9 250.x1 or 250.x3).

1) Identification of patients who already have a T2D diagnosis:

    a) Include patients with Type 2 Diabetes diagnosis based on ICD9 code (excluding those with ketoacidosis codes).

    b) Exclude patients (currently) treated only with insulin AND have never been on a type 2 diabetes medication, and: diagnosed with T1D, or even if not diagnosed with T1D, diagnosed with T2D on < 2 dates in an encounter or problem list.

2) Identification of patients who do not yet have a T2D diagnosis: Include patients with haemoglobin A1C lab value ≥ 6.5%, fasting glucose > 125 mg/dl or random glucose > 200 mg/dl AND prescribed one of the medications (or combinations thereof)

sulfonylureas, meglitinides, biguanides, thiazoldinediones, alpha-glycosidase inhibitors, DPPIV inhibitor and injectable.

Control selection criteria:

a) Have had at least 2 clinic visits (face-to-face outpatient clinic encounters).

b) Have not been assigned an ICD9 code for diabetes (type 1 or type 2) or any diabetes-related condition.

c) Have not been prescribed insulin or Pramlintide, or any medications for diabetes treatment, or diabetic supplies such as those for medication administration or glucose monitoring.

d) Do not have a reported (random or fasting) blood glucose ≥ 110mg/dl and have had at least 1 glucose measurement.

e) Do not have a reported haemoglobin A1c ≥ 6.0%.

f) Do not have a reported family history of diabetes (type 1 or type 2).

FUSION

dbGaP Study Accession: phs000100.v4.p1
Ethnicity: European (Finland)
T2D cases after QC: 901
Controls after QC: 772

Type 2 diabetes case selection criteria:

a) 644 FUSION and 275 Finrisk 2002 T2D cases as defined by WHO 1999 criteria of fasting plasma glucose ≥ 7.0 mmol/l or 2-h plasma glucose ≥ 11.1 mmol/l, by report of diabetes medication use, or based on medical record review.

b) FUSION cases with known or probable T1D among their first-degree relatives were excluded.

c) The 644 FUSION cases reported at least one T2D sibling.

d) The Finrisk cases came from a Finnish population-based risk factor survey.

Control selection criteria:

a) 331 FUSION and 456 Finrisk 2002 NGT controls as defined by WHO 1999 criteria of fasting glucose < 6.1 mmol/l and 2-h glucose < 7.8 mmol/l.

b) FUSION controls include 119 subjects from Vantaa, Finland, who were NGT at ages 65 and 70 years, and 212 NGT spouses of FUSION subjects. The controls were approximately frequency matched to the cases by age, sex, and birth province.

GENEVA Genes and Environment Initiatives in Type 2 Diabetes (Nurses' Health Study/Health Professionals Follow-up Study) GENEVA NHS/HPFS

dbGaP Study Accession: phs000091.v2.p1
Ethnicity: European (USA)
T2D cases after QC: 2614
Controls after QC: 3061

Type 2 diabetes case selection criteria:

Through 1996 follow-up, criteria for confirmed T2D included one of the following:

a) One or more classic symptoms (excessive thirst, polyuria, weight loss, hunger, pruritus, or coma) plus fasting plasma glucose ≥ 140 mg/dl (7.8 mmol/L) and/or random plasma glucose ≥ 200 mg/dl (11.1 mmol/L) and/or plasma glucose 2 hours after an oral glucose tolerance test ≥ 200 mg/dl; or

b) At least two elevated plasma glucose levels (as described above) on different occasions in the absence of symptoms; or

c) Treatment with hypoglycaemic medication (insulin or oral hypoglycaemic agent).

In response to the current ADA diagnostic criteria (fasting plasma glucose cut point ≥ 126 mg/dl [7.0 mmol/L]), Supplementary Diabetes Questionnaire for participants reporting a new diagnosis of diabetes on the 1998 or later questionnaires were revised. This revised supplementary questionnaire ascertains the level of elevation in fasting plasma glucose and facilitates determining which participants had fasting plasma glucose ≥ 140 mg/dl (the earlier diagnostic cut point) and which had a fasting plasma glucose ≥ 126 (the current diagnostic cut point). The criteria for confirmed T2D during the 1998–2000 follow-up cycle and later cycles remain the same, except for the elevated fasting plasma glucose criterion for which the cut point was changed from 140 mg/dl to 126 mg/dl. The revised supplementary questionnaire was used to classify cases in categories of glucose elevation and determine the proportion diagnosed in each category (e.g. fasting plasma glucose 126–139 versus ≥ 140 mg/dl) allowing conducting sensitivity analyses with exclusion of participants that meet the ADA criteria and not the NDDG criteria.

Control selection criteria: No diabetes mellitus.

Wellcome Trust Case Control Consortium (WTCCC)

EGA Study ID: EGAS00000000005 (EGAS00000000001 + EGAS00000000002 + EGAS00000000009)

Ethnicity: European (UK)

T2D cases after QC: 1894

Controls after QC: 2917

T2D case selection criteria:

The T2D cases were selected from UK Caucasian subjects who form part of the Diabetes UK Warren 2 repository. In each case, the diagnosis of diabetes was based on either current prescribed treatment with sulphonylureas, biguanides, other oral agents and/or insulin or, in the case of individuals treated with diet alone, historical or contemporary laboratory evidence of hyperglycaemia (as defined by the World Health Organization). Other forms of diabetes (for example, maturity-onset diabetes of the young, mitochondrial diabetes, and T1D) were excluded by standard clinical criteria based on personal and family history. Criteria for excluding autoimmune diabetes included absence of first-degree relatives with T1D, an interval of ≥1 years between diagnosis and institution of regular insulin therapy and negative testing for antibodies to glutamic acid decarboxylase (anti-GAD). Cases were limited to those who reported that all four grandparents had exclusively British and/or Irish origin, by both self-reported ethnicity and place of birth. All were diagnosed between age 25 and 75. Approximately 30% were explicitly recruited as part of multiplex sibships[2] and ~25% were offspring in parent-offspring 'trios' or 'duos' (that is, families comprising only one parent complemented by additional sibs)[3]. The remainders were recruited as isolated cases but these cases were (compared to population-based cases) of relatively early onset and had a high proportion of T2D parents and/or siblings[4]. Cases were ascertained across the UK but were centralized on the main collection centres (Exeter, London, Newcastle, Norwich, Oxford). Selection of the samples typed in WTCCC from the larger collections was based primarily on DNA availability and success in passing Diabetes and Inflammation Laboratory (DIL)/Wellcome Trust Sanger Institute (WTSI) DNA quality control.

Control selection criteria:

a) The 1958 Birth Cohort (also known as the National Child Development Study) includes all births in England, Wales and Scotland, during one week in 1958. From an original sample of over 17,000 births, survivors were followed up at ages 7, 11,

16, 23, 33 and 42 years (http://www.cls.ioe.ac.uk/studies.asp?section=000100020003). In a biomedical examination at 44-45 years[5] (http://www.b58cgene.sgul.ac.uk/followup.php), 9,377 cohort members were visited at home providing 7,692 blood samples with consent for future Epstein-Barr virus (EBV)-transformed cell lines. DNA samples extracted from 1,500 cell lines of self-reported white ethnicity and representative of gender and each geographical region were selected for use as controls.

b) The second set of common controls was made up of 1,500 individuals selected from a sample of blood donors recruited as part of the current project. WTCCC in collaboration with the UK Blood Services (NHSBT in England, SNBTS in Scotland and WBS in Wales) set up a UK national repository of de-identified samples of DNA and viable mononuclear cells from 3,622 consenting blood donors, age range 18-69 years (ethical approval 05/Q0106/74). A set of 1,564 samples was selected from the 3622 samples recruited based on sex and geographical region (to reproduce the distribution of the samples of the 1958 Birth Cohort) for use as common controls in the WTCCC study. DNA was extracted as described in the original WTCCC study[6] with a yield of 3054 ± 1207 μg (mean ± 1 s.d.).

Genetic Epidemiology Research on Adult Health and Aging (GERA)

dbGaP Study Accession: phs000674.v1.p1
Ethnicity: European (USA)
T2D cases after QC: 6995
Controls after QC: 49845

Inclusion criteria:

a) Eligible for RPGEH survey

a) ≥ 18 years of age at time of survey mailing (2007).

b) KP Northern California Region enrolee for at least 2 years prior to survey.

b) Consented to contribute biospecimen to RPGEH and returned saliva sample by cut-off date for GERA genotyping.

c) All available samples from minorities were included, plus Non-Hispanic Whites selected at random to reach 110,266 participants with extracted DNA whose samples were submitted for genotyping.

d) Successfully genotyped (DQC ≥ 0.82; call rate ≥ 0.97) from extracted DNA.

e) Consented explicitly to have data deposited in NIH-maintained database.

Exclusion criteria:

a) Subject requested withdrawal from study after DNA extraction and genotyping.

b) Validity of link between biospecimen and study participant questionable because of genotype-phenotype discordance, e.g. gender.

A participant was coded as a T2D patient if he/she had at least two diagnoses within this disease category that had to be recorded on separate days. Diagnoses were obtained from patient encounters at Kaiser Permanente Northern California facilities from January 1, 1995 to March 15, 2013. The March 2013 ICD9-CM diagnoses used for the Type 2 Diabetes category were:

a) 250.00 Diabetes mellitus without mention of complication, type II or unspecified type, not stated as uncontrolled.

b) 250.02 Diabetes mellitus without mention of complication, type II or unspecified type, uncontrolled.

c) 250.10 Diabetes with ketoacidosis, type II or unspecified type, not stated as uncontrolled.
d) 250.12 Diabetes with ketoacidosis, type II or unspecified type, uncontrolled.
e) 250.20 Diabetes with hyperosmolarity, type II or unspecified type, not stated as uncontrolled.
f) 250.22 Diabetes with hyperosmolarity, type II or unspecified type, uncontrolled.
g) 250.30 Diabetes with other coma, type II or unspecified type, not stated as uncontrolled.
h) 250.32 Diabetes with other coma, type II or unspecified type, uncontrolled.
i) 250.40 Diabetes with renal manifestations, type II or unspecified type, not stated as uncontrolled.
j) 250.42 Diabetes with renal manifestations, type II or unspecified type, uncontrolled.
k) 250.50 Diabetes with ophthalmic manifestations, type II or unspecified type, not stated as uncontrolled.
l) 250.52 Diabetes with ophthalmic manifestations, type II or unspecified type, uncontrolled.
m) 250.60 Diabetes with neurological manifestations, type II or unspecified type, not stated as uncontrolled.
n) 250.62 Diabetes with neurological manifestations, type II or unspecified type, uncontrolled.
o) 250.70 Diabetes with peripheral circulatory disorders, type II or unspecified type, not stated as uncontrolled.
p) 250.72 Diabetes with peripheral circulatory disorders, type II or unspecified type, uncontrolled.

q) 250.80 Diabetes with other specified manifestations, type II or unspecified type, not stated as uncontrolled.

r) 250.82 Diabetes with other specified manifestations, type II or unspecified type, uncontrolled.

s) 250.90 Diabetes with unspecified complication, type II or unspecified type, not stated as uncontrolled.

t) 250.92 Diabetes with unspecified complication, type II or unspecified type, uncontrolled.

The rest of subjects not coded as T2D patients were considered as controls.

DIAGRAM Trans-Ethnic meta-analysis

We used the summary statistics for the trans-ethnic T2D GWAS meta-analysis[7] from the DIAGRAM consortium, which comprises the following ancestry-specific meta-analyses: the DIAGRAM Consortium (12,171 cases and 56,862 controls, European ancestry); the AGEN-T2D Consortium (6,952 cases and 11,865 controls, East Asian ancestry); the SAT2D Consortium (5,561 cases and 14,458 controls, South Asian ancestry); and the MAT2D Consortium (1,804 cases and 779 controls, Mexican and Mexican American ancestry). Each individual study undertook sample and SNP quality control (QC), and the genomic resolution was increased up to 2.5 million autosomal SNPs thanks to genotype imputation with Phase II/III HapMap reference panel. QCed SNPs with MAF>1%, (except MAF>5% in the Mexican and Mexican American ancestry GWAS due to smaller sample size) were tested for association with T2D under an additive model adjusted for several study specific covariates. Association summary statistics were combined via fixed-effects according to the ancestry group, and the results of each ancestry-specific meta-analysis were combined thanks to a

fixed effects inverse-variance weighted meta-analysis, comprising a total sample size of 26,488 cases and 83,964 controls.

Type 2 Diabetes Knowledge Portal (T2D Portal)

The T2D Portal (http://www.type2diabetesgenetics.org/) is a central repository for obtaining summary statistics from large genetic association studies of T2D, including projects based on whole-exome sequencing data or exome arrays for low-frequency data and SNP arrays covering common variation (GWAS). Besides this, T2D Knowledge Portal has also included the results from GWAS meta-analysis of 24 other traits.

In our study we used the summary statistics from whole-exome sequencing analysis and exome chip analysis. First, the summary statistics of 16,857 individual exome sequences were derived from the integration of multiple projects such as T2D-GENES, GoT2D and SIGMA. This dataset comprises individuals from 5 ethnic groups (African-American, East Asian, South Asian, European and Hispanic)[8,9]. Additionally, we also used the summary statistics from exome chip analysis of 75,670 individuals from European ancestry. This dataset has integrated the efforts from the DIAGRAM consortium, the GoT2D project and the T2D-GENES project[8]. This data was accessed on June 2016.

Supplementary Note 2

Summary of replication datasets for the rs146662075 X-chromosome variant

InterAct

The InterAct consortium[10] entails a case-cohort study that aroused from the existing large cohort 'EPIC' study. The EPIC study comprises 350,000 participants from 10 European countries and a lot of effort was put in standardizing lifestyle and dietary information. After a

follow-up of 8 years, T2D has been diagnosed to 12,403 individuals and InterAct has also defined a cohort of 16,154 controls free of diabetes at baseline. Participants in epic interact provided informed consent. This study was approved by each centre ethics committee and the International Agency for Research on Cancer, the coordinating body for EPIC Europe.

From the SNP and sample QCed data, we extracted the male samples corresponding to 6,763 individuals, which were re-analysed using genotype imputation with the UK10K reference panel. Association with T2D has been evaluated using an additive logistic model with SNPTEST v2.5.2 adjusted by age and body-mass index.

Slim Initiative in Genomic Medicine for the Americas (SIGMA) T2D Genetics Consortium

The SIGMA consortium GWAS dataset comprised of 8,214 individuals (3,848 T2D cases and 4,366 controls), consisting of four independent cohorts of Mexican or individuals with Latin American ancestry; The Diabetes in Mexico Study (DMS), Mexico City Diabetes Study (MCDS), Multiethnic Cohort (MEC) and UNAM/INCMNSZ Diabetes (UIDS) cohorts[11]. Genotyping of study participants using the Illumina OMNI2.5 array have been described previously[11]. These cohorts, after the SNP and sample QC, were imputed using the UK10K reference panel and the association with T2D was tested under an additive logistic model only considering male samples with SNPTEST v2.5.2 adjusted by age and body-mass index.

Danish cohort

The Danish replication data consisted of data from five sample sets: 1) Inter99, a population-based randomized controlled trial (CT00289237, ClinicalTrials.gov) investigating the effects of lifestyle intervention on cardiovascular disease[12]; 2) Health2006 cohort, a population-based epidemiological study of general health, diabetes and cardiovascular disease[13]; 3) ADDITION-DK screening cohort, 4) Vejle Biobank diabetes case-control study; and 5) clinical type 2 diabetes cases ascertained at Steno Diabetes Center.

All individuals were of Danish nationality. Written informed consent was obtained from all participants. The studies were approved by the local Scientific Ethics Committees and were performed in accordance with the principles of the Declaration of Helsinki II.

T2D was defined according to WHO 1999 criteria. Control individuals had fasting plasma glucose < 6.1 mmol/L (all study groups) and furthermore 2 hr plasma glucose during an oral glucose tolerance test < 7.8 mmol/l (study group 1). For the case-control analysis, we defined three definitions of controls: a) Any subject with fasting plasma glucose <6.1 mmol/L; b) any subject with fasting plasma glucose <6.1 and older than 55 years (which corresponds do average age at onset of T2D); c) any subject older than 55 and oral glucose tolerant test values below 7.8 mmol/l (study group 1).

Genotyping of Danish samples was performed by KASPar SNP Genotyping System (LGC Genomics, Hoddeson, UK). Ten selected samples from the 1000 Genomes Project (Coriell) were genotyped together with the study samples to estimate mismatch between genotyping and sequencing. All genotypes (5 heterozygous and 5 homozygous for reference allele) were concordant. Furthermore, 1,602 study samples were genotyped in duplicate and no mismatches were observed. Moreover, general call rate was 98%. Genotype distribution was in accordance with Hardy-Weinberg equilibrium.

The Kaplan-Meier method was used to plot cumulative incidence of T2D against time of follow-up in the Inter99 cohorts, which were followed for 11 years on average. Cox proportional hazards regression models were used to address the risk of incident T2D. Individuals with self-reported diabetes at the baseline examination and individuals present in the Danish National Diabetes Registry before the baseline examination were excluded from the present analyses of incident T2D. The follow-up analysis were restricted to male

individuals younger than 45 years old, which will reach 56 years old after 11 years of follow-up.

Partners Biobank

The Partners HealthCare Biobank[14] maintains blood and DNA samples from more than 60,000 consented patients seen at Partners HealthCare hospitals, including Massachusetts General Hospital, Brigham and Women's Hospital, McLean Hospital, and Spaulding Rehabilitation Hospital, all in the USA. Patients are recruited in the context of clinical care appointments at more than 40 sites, clinics and also electronically through the patient portal at Partners HealthCare. Biobank subjects provide consent for the use of their samples and data in broad-based research. The Partners Biobank works closely with the Partners Research Patient Data Registry (RPDR), the Partners' enterprise scale data repository designed to foster investigator access to a wide variety of phenotypic data on more than 4 million Partners HealthCare patients. The approval for analysis of Biobank data was obtained by Partners IRB, study 2016P001018.

Type 2 diabetes status was defined based on "curated phenotypes" developed by the Biobank Portal team using both structured and unstructured electronic medical record (EMR) data and clinical, computational and statistical methods. Natural Language Processing (NLP) was used to extract data from narrative text. Chart reviews by disease experts helped identify features and variables associated with particular phenotypes and were also used to validate results of the algorithms. The process produced robust phenotype algorithms that were evaluated using metrics such as sensitivity, the proportion of true positives correctly identified as such, and positive predictive value (PPV), the proportion of individuals classified as cases by the algorithm[15].

a) Control selection criteria.

1) Individuals determined by the "curated disease" algorithm employed above to have no history of type 2 diabetes with NPV of 99%.
2) Individuals at least age 55.
3) Individuals with HbA1c less than 5.7

b) Case selection criteria.

1) Individuals determined by the "curated disease" algorithm employed above to have type 2 diabetes with PPV of 99%
2) Individuals at least age 30 given the higher rate of false positive diagnoses in younger individuals.

Genomic data for 15,061 participants was generated with the Illumina Multi-Ethnic Genotyping Array, which covers more than 1.7 million markers, including content from over 36,000 individuals, and is enriched for exome content with >400,000 markers missense, nonsense, indels, and synonymous variants.

UK Biobank

The UK Biobank is a prospective cohort of ~500,000 individuals aged between 40 to 69 years when recruited in 2006-2010[16]. Participants agreed to provide detailed information about their lifestyle, environment and medical history, biological samples (for genotyping and for biochemical assays), to undergo measures and to have their health followed (http://www.ukbiobank.ac.uk/). The UK Biobank has obtained ethical approval covering this study from the National Research Ethics Committee (REC reference 11/NW/0382).

As the current UK Biobank data release did not include imputed data for the X Chromosome, phasing and imputation was run in house. The data release used in this work comprises X chromosome QCed genotypes of 488,377 UK Biobank participants, which were assayed using two arrays sharing 95% of marker content (Applied Biosystems™ UK BiLEVE

Axiom™ Array and the Applied Biosystems™ UK Biobank Axiom™ Array)[17]. Before phasing the X chromosome genotypes into haplotypes, we performed additional QC analysis. First, we used information already provided by UK Biobank: we included those samples that were used as input for phasing and without excess of relatedness. We also included all markers used as input for phasing and present in both arrays after the UK Biobank QC. Then, we performed an additional sample QC by excluding women, individuals with missing rate > 5% or showing gender discordance between the reported and the genetically predicted sex. At the variant level, we excluded those markers with MAF < 0.1% and with a missingness rate > 5%. The resulting dataset comprised 16,463 markers and 222,725 male individuals. Due to the huge computational burden, we split the cohort in 6 homogenous subsets. To do that, we prioritized keeping all the individuals genotyped by the UK BiLEVE array in a single subset and we also respected the different batches defined by UK Biobank. We performed a two-stage imputation procedure based on first pre-phasing the genotypes into whole chromosome haplotypes followed by genotype imputation with the UK10K reference panel (http://www.uk10k.org/). Phasing was performed with SHAPEIT2 and the IMPUTE2 software was used for genotype imputation. During the imputation step we excluded *indels*, variants whose pairs of alleles were either A/T or C/G, variants with MAF < 1% and variants showing deviation of Hardy-Weinberg Equilibrium with $p < 1x10^{-20}$. In addition, from those pairs of relatives reported to be third-degree or higher according to UK Biobank, we excluded from each pair the individual with lowest call rate. We tested the rs146662075 variant for association with SNPTEST_v2.5.1 using the *threshold* method and including 7 principal components, body mass index (BMI), age at recruitment and batch information as covariates. To build our case-control analysis we used the following criteria:

a) Control selection criteria.

(1) Individuals without any primary or secondary ICD-10 diagnose from hospitalization events included in the following disease categories: E10 (Insulin-dependent diabetes mellitus), E11 (Non-insulin-dependent diabetes mellitus), E13 (Other specified diabetes mellitus) and E14 (Unspecified diabetes mellitus).

(2) Individuals without family history of diabetes mellitus (father, mother or siblings).

(3) Individuals whose age at recruitment ≥ 55 years old.

(4) Individuals without reported age at onset of diabetes mellitus.

b) Case selection criteria.

(1) Individuals with a primary or secondary ICD-10 diagnose from hospitalization events included in the E11 (Non-insulin-dependent diabetes mellitus) disease category.

(2) Individuals without any primary or secondary ICD-10 diagnose from hospitalization events included in the following disease categories: E10 (Insulin-dependent diabetes mellitus), E13 (Other specified diabetes mellitus) and E14 (Unspecified diabetes mellitus).

Finally, we used inverse variance fixed effect meta-analysis to obtain the final effect-size, standard error and p-value across the association results from each of the 6 subsets.

Supplementary Note 3

The SIGMA Type 2 Diabetes Genetics Consortium

Genetic analyses:

Josep M. Mercader[1, 2, 3], Alicia Huerta-Chagoya[4], Humberto García-Ortiz[5], Hortensia Moreno-Macías[4, 6], Alisa Manning[3, 7, 8], Lizz Caulkins[3], Noël P. Burtt[3], Jason Flannick[3, 9], Nick Patterson[10], Carlos A. Aguilar-Salinas[4], Teresa Tusié-Luna[4, 11], David Altshuler[3, 9, 12], Jose C. Florez[1, 3, 8, 13]

Study cohorts:

Diabetes in Mexico Study: Humberto García-Ortiz[5], Angélica Martínez-Hernández[5], Federico Centeno-Cruz[5], Francisco Martin Barajas-Olmos[5], Carlos Zerrweck[14], Cecilia Contreras-Cubas[5], Elvia Mendoza-Caamal[5], Cristina Revilla-Monsalve[15], Sergio Islas-Andrade[15], Emilio Córdova[5], Xavier Soberón[5], Lorena Orozco[5]

Mexico City Diabetes Study: Clicerio González-Villalpando[16], María Elena González-Villalpando[16]

Multiethnic Cohort Study: Christopher A. Haiman[17], Lynne Wilkens[18], Loic Le Marchand[18], Kristine Monroe[17], Laurence Kolonel[18]

UNAM/INCMNSZ Diabetes Study: Olimpia Arellano-Campos[4], Alicia Huerta-Chagoya[4], Maria L. Ordóñez-Sánchez[4], Maribel Rodríguez-Torres[4], Yayoi Segura-Kato[4], Rosario Rodríguez-Guillén[4], Ivette Cruz-Bautista[4], Linda Liliana Muñoz-Hernandez[4], Tamara Sáenz[4], Donají Gómez[4], Ulices Alvirde[4], Paloma Almeda-Valdés[4], Hortensia Moreno-Macías[4, 6], Teresa Tusié-Luna[4, 11], Carlos A. Aguilar-Salinas[4]

Scientific and project management:

Noël P. Burtt[3], Lizz Caulkins[3], Maria L. Cortes[10]

Steering committee:

David Altshuler[3, 9, 12], Jose C. Florez[1, 3, 8, 13], Christopher A. Haiman[17], Carlos A. Aguilar-Salinas[4], Clicerio González-Villalpando[16], Lorena Orozco[5], Teresa Tusié-Luna[4, 11]

[1] Diabetes Unit and Center for Human Genetic Research, Massachusetts General Hospital, Boston, Massachusetts, 02114, USA.

[2] Joint BSC-CRG-IRB Research Program in Computational Biology. Barcelona Supercomputing Center, 08034 Barcelona.

[3] Program in Medical and Population Genetics, Broad Institute of Harvard and MIT, Cambridge, Massachusetts, 02142, USA.

[4] Instituto Nacional de Ciencias Médicas y Nutrición Salvador Zubirán, Sección XVI, Tlalpan, 14000 Mexico City, Mexico.

[5] Instituto Nacional de Medicina Genómica, Tlalpan, 14610, Mexico City, Mexico.

[6] Universidad Autónoma Metropolitana, Tlalpan 14387, Mexico City, Mexico.

[7] Center for Human Genetic Research, Massachusetts General Hospital, Boston, Massachusetts, 02114, USA.

[8] Department of Medicine, Harvard Medical School, Boston, Massachusetts, USA.

[9] Department of Molecular Biology, Massachusetts General Hospital, Boston, Massachusetts, 02114, USA.

[10] Broad Institute of Harvard and MIT, Cambridge, Massachusetts, 02142, USA.

[11] Instituto de Investigaciones Biomédicas, UNAM Unidad de Biología Molecular y Medicina Genómica, UNAM/INCMNSZ, Coyoacán, 04510 Mexico City, Mexico.

[12] Department of Genetics, Harvard Medical School, Boston, Massachusetts, 02115, USA.

[13] Metabolism Program, Broad Institute of Harvard and MIT, Cambridge, Massachusetts, 02142, USA.

[14] Clínica de Integral de Cirugía para la Obesidad y Enfermedades Metabólicas, Hospital General Tláhuac, Secretaría de Salud del GDF. México City.

[15] Instituto Mexicano del Seguro Social SXXI, Mexico City, Mexico.

[16] Centro de Estudios en Diabetes, Unidad de Investigacion en Diabetes y Riesgo Cardiovascular, Centro de Investigacion en Salud Poblacional, Instituto Nacional de Salud Publica, Mexico City, Mexico.

[17] Department of Preventive Medicine, Keck School of Medicine, University of Southern California, Los Angeles, California, 90033, USA.

[18] Epidemiology Program, University of Hawaii Cancer Center, Honolulu, Hawaii, USA.

Supplementary Note 4

InterAct Consortium

Claudia Langenberg[1], Robert A. Scott[1], Stephen J. Sharp[1], Nita G. Forouhi[1], Nicola D. Kerrison[1], Matt Sims[1], Debora ME Lucarelli[1], Inês Barroso[2,3], Panos Deloukas[2], Mark I. McCarthy[4,5,6], Larraitz Arriola[7,8,9], Beverley Balkau[10,11], Aurelio Barricarte[9,12,13], Heiner Boeing[14], Paul W. Franks[15,16], Carlos Gonzalez[17], Sara Grioni[18], Rudolf Kaaks[19], Timothy J. Key[20], Carmen Navarro[9,21,22], Peter M. Nilsson[15], Kim Overvad[23,24], Domenico Palli[25], Salvatore Panico[26], J. Ramón Quirós[27], Olov Rolandsson[16], Carlotta Sacerdote[28,29], María-José Sánchez[9,30,31], Nadia Slimani[32], Anne Tjonneland[33], Rosario Tumino[34], Daphne L. van der A[35], Yvonne T. van der Schouw[36], Elio Riboli[37], Nicholas J. Wareham[1]

Affiliations:
[1] MRC Epidemiology Unit, University of Cambridge, Cambridge, United Kingdom.
[2] The Wellcome Trust Sanger Institute, Cambridge, United Kingdom.
[3] University of Cambridge Metabolic Research Laboratories, Cambridge, UK.
[4] Oxford Centre for Diabetes, Endocrinology and Metabolism (OCDEM), University of Oxford, UK.
[5] Wellcome Trust Centre for Human Genetics, University of Oxford, Oxford, UK.
[6] Oxford NIHR Biomedical Research Centre, Oxford, UK.
[7] Public Health Division of Gipuzkoa, San Sebastian, Spain.
[8] Instituto BIO-Donostia, Basque Government, San Sebastian, Spain.
[9] CIBER Epidemiología y Salud Pública (CIBERESP), Spain.
[10] Inserm, CESP, U1018, Villejuif, France.
[11] Univ Paris-Sud, UMRS 1018, Villejuif, France.
[12] Navarre Public Health Institute (ISPN), Pamplona, Spain.
[13] Navarra Institute for Health Research (IdiSNA) Pamplona, Spain.
[14] German Institute of Human Nutrition Potsdam-Rehbruecke, Germany.

[15] Lund University, Malm÷, Sweden.
[16] Umeσ University, Umeσ, Sweden.
[17] Catalan Institute of Oncology (ICO), Barcelona, Spain.
[18] Epidemiology and Prevention Unit, Milan, Italy.
[19] German Cancer Research Centre (DKFZ), Heidelberg, Germany.
[20] University of Oxford, United Kingdom.
[21] Department of Epidemiology, Murcia Regional Health Council, IMIB-Arrixaca, Murcia, Spain.
[22] Unit of Preventive Medicine and Public Health, School of Medicine, University of Murcia, Spain.
[23] Department of Public Health, Section for Epidemiology, Aarhus University, Aarhus, Denmark.
[24] Aalborg University Hospital, Aalborg, Denmark.
[25] Cancer Research and Prevention Institute (ISPO), Florence, Italy.
[26] Dipartimento di Medicina Clinica e Chirurgia, Federico II University, Naples, Italy.
[27] Public Health Directorate, Asturias, Spain.
[28] Unit of Cancer Epidemiology, Citta' della Salute e della Scienza Hospital-University of Turin and Center for Cancer Prevention (CPO), Torino, Italy.
[29] Human Genetics Foundation (HuGeF), Torino, Italy.
[30] Andalusian School of Public Health, Granada, Spain.
[31] Instituto de Investigación Biosanitaria de Granada (Granada.ibs), Granada (Spain).
[32] International Agency for Research on Cancer, Lyon, France.
[33] Danish Cancer Society Research Center, Copenhagen, Denmark.
[34] ASP Ragusa, Italy.
[35] National Institute for Public Health and the Environment (RIVM), Bilthoven, The Netherlands.
[36] University Medical Center Utrecht, Utrecht, the Netherlands, (37) School of Public Health, Imperial College London, UK.

Supplementary References

1. Pasquali, L. *et al.* Pancreatic islet enhancer clusters enriched in type 2 diabetes risk-associated variants. *Nat Genet* **46**, 136-43 (2014).
2. Wiltshire, S. *et al.* A genomewide scan for loci predisposing to type 2 diabetes in a U.K. population (the Diabetes UK Warren 2 Repository): analysis of 573 pedigrees provides independent replication of a susceptibility locus on chromosome 1q. *Am J Hum Genet* **69**, 553-69 (2001).
3. Frayling, T.M. *et al.* Parent-offspring trios: a resource to facilitate the identification of type 2 diabetes genes. *Diabetes* **48**, 2475-9 (1999).
4. Groves, C.J. *et al.* Association analysis of 6,736 U.K. subjects provides replication and confirms TCF7L2 as a type 2 diabetes susceptibility gene with a substantial effect on individual risk. *Diabetes* **55**, 2640-4 (2006).
5. Strachan, D.P. *et al.* Lifecourse influences on health among British adults: effects of region of residence in childhood and adulthood. *Int J Epidemiol* **36**, 522-31 (2007).
6. Wellcome Trust Case Control, C. Genome-wide association study of 14,000 cases of seven common diseases and 3,000 shared controls. *Nature* **447**, 661-78 (2007).
7. DIAbetes Genetics Replication And Meta-analysis (DIAGRAM) Consortium *et al.* Genome-wide trans-ancestry meta-analysis provides insight into the genetic architecture of type 2 diabetes susceptibility. *Nat Genet* **46**, 234-44 (2014).
8. Fuchsberger, C. *et al.* The genetic architecture of type 2 diabetes. *Nature* **536**, 41-47 (2016).
9. Consortium, S.T.D. *et al.* Association of a low-frequency variant in HNF1A with type 2 diabetes in a Latino population. *JAMA* **311**, 2305-14 (2014).

10. Langenberg, C. *et al.* Gene-lifestyle interaction and type 2 diabetes: the EPIC interact case-cohort study. *PLoS Med* **11**, e1001647 (2014).
11. Consortium, S.T.D. *et al.* Sequence variants in SLC16A11 are a common risk factor for type 2 diabetes in Mexico. *Nature* **506**, 97-101 (2014).
12. Jorgensen, T. *et al.* A randomized non-pharmacological intervention study for prevention of ischaemic heart disease: baseline results Inter99. *Eur J Cardiovasc Prev Rehabil* **10**, 377-86 (2003).
13. Thuesen, B.H. *et al.* Cohort Profile: the Health2006 cohort, research centre for prevention and health. *Int J Epidemiol* **43**, 568-75 (2014).
14. Karlson, E.W., Boutin, N.T., Hoffnagle, A.G. & Allen, N.L. Building the Partners HealthCare Biobank at Partners Personalized Medicine: Informed Consent, Return of Research Results, Recruitment Lessons and Operational Considerations. *J Pers Med* **6**(2016).
15. Yu, S. *et al.* Toward high-throughput phenotyping: unbiased automated feature extraction and selection from knowledge sources. *J Am Med Inform Assoc* **22**, 993-1000 (2015).
16. Sudlow, C. *et al.* UK biobank: an open access resource for identifying the causes of a wide range of complex diseases of middle and old age. *PLoS Med* **12**, e1001779 (2015).
17. Bycroft, C. *et al.* Genome-wide genetic data on ~500,000 UK Biobank participants. *bioRxiv* (2017).
