# Supplementary material for: Re-analysis of public genetic data reveals a rare X-chromosomal variant associated with type 2 diabetes
Source: Nat Commun. 2018 Jan 22;9:321. doi: 10.1038/s41467-017-02380-9 (PMC5778074; doi:10.1038/s41467-017-02380-9)
Supplement: Supplementary file 3 — Description of Additional Supplementary Information [file 41467_2017_2380_MOESM3_ESM.docx]

**Description of Additional Supplementary Files**

File Name: Supplementary Data 1

Description: Description of T2D cohorts included in the meta-analysis

File Name: Supplementary Data 2

Description: Tissue enrichment of genes at Type II diabetes associated loci (P-value<1e-05, FDR<=0.20)

File Name: Supplementary Data 3

Description: Gene set enrichment of genes at Type II Diabetes associated loci (P-value<1e-05, FDR<=0.05). The results are organized as clusters of pathways according to their similarity. The top 10 genes driving the enrichment and their z-scores are also shown.

File Name: Supplementary Data 4

Description: Results from previously described variants in the 70KforT2D meta-analysis.

File Name: Supplementary Data 5

Description: 99% credible sets generated with the 70KforT2D discovery dataset. Credible sets were generated for all the regions that reached GWAS significance by meta-analyzing the 70kforT2D and their suitable replication cohorts (see Figure 1).

File Name: Supplementary Data 6

Description: Structural variants within the 99% credible sets of all the identified loci. The table describes wether this variant was detected in 1000G phase 1 or in UK10K reference panel and wethere this variant was also confirmed in 1000G phase3.

File Name: Supplementary Data 7

Description: Variants with moderate or high annotation according to VEP.

File Name: Supplementary Data 8

Description: Combined Annotation Dependent Depletion (CADD) for variants in the 99% credible sets. Prediction of deleteriousness for coding and non-coding variants. Only variants with CADD higher than 10 are shown.

File Name: Supplementary Data 9

Description: Probability of Fitness Consequences according to LINSIGHT for 99% credible sets generated with the 70KforT2D discovery dataset.

File Name: Supplementary Data 10

Description: Variants within the 99% credible sets that show significant gene-wide significant eQTLs (based on permutations) in any given tissue in GTEx (V6). For each variant, all the associations in all tissues that achieved significance q-value below 0.05 are represented. eQTLs mapping to HLA genes have been excluded as alignments in these regions can be problematic.

File Name: Supplementary Data 11

Description: Variants within the 99% credible sets that show significant gene-wide significant eQTLs, sQTLs, and allelic imbalance in pancreatic islets (Fadista et al. 2014, PNAS). For each variant, all the associations with all tissues is represented. eQTLs mapping to HLA genes have been excluded as alignments in these regions can be problematic.

File Name: Supplementary Data 12

Description: Conditional Analysis for rs3794205 variant leading the novel CAMKK2 association and T2D signals from the HN1FA locus.

File Name: Supplementary Data 13

Description: Conditional Analysis for the rs115884658 variant leading the novel EHMT2 association, the lead variant from the 99% credible set and T2D and T1D leading variants from the MHC region.

File Name: Supplementary Data 14

Description: Conditional Analysis for the rs5943526 variant reported to lead the DUSP9 locus and rs61503151, novel lead variant for the same region found in the 70KforT2D meta-analysis .
